# Investigating the accuracy, risk impact, and cost-effectiveness of component-resolved diagnostic test for food allergy: a systematic review protocol

**DOI:** 10.1038/s41533-017-0015-0

**Published:** 2017-02-09

**Authors:** Javier Flores Kim, Bright I. Nwaru, Nicola McCleary, Andrew Stoddart, Aziz Sheikh

**Affiliations:** 1 Asthma UK Centre for Applied Research, Centre for Medical Informatics, Usher Institute of Population Health Sciences and Informatics, The University of Edinburgh, Edinburgh, UK; 2 School of Health Sciences, University of Tampere, Tampere, Finland; 3 Edinburgh Clinical Trials Unit, Centre for Medical Informatics, Usher Institute of Population Health Sciences and Informatics, The University of Edinburgh, Edinburgh, EH8 9AG UK

## Background

Food allergy—particularly immunoglobulin E (IgE)-mediated food allergy—is now a global public health problem. It results in considerable morbidity, healthcare utilisation, and impairment in quality of life.^1–6^ Food allergy reactions range in severity from relatively mild features to life-threatening anaphylaxis.^2, 3^ Estimates of the prevalence of food allergy vary, but overall lifetime prevalence has been estimated as affecting up to 7% of children and 6% of adults.^4, 5^

Careful avoidance of offending foods is the mainstay preventive strategy for food allergy.^2, 3^ Accurate diagnosis is essential in order to provide appropriate, potentially life-saving advice on how to prevent and manage reactions. This will also help to prevent unwarranted dietary restrictions.^2, 3^ The diagnosis of food allergy is typically based on a combination of clinical history and an objective marker of allergic sensitisation, namely, skin prick tests (SPT) and/or immunoassays of serum-specific IgE levels. While these approaches have reasonable sensitivity, they have sub-optimal specificity and are in general poorly predictive of the severity of reactions. Further diagnostic confirmation with a double-blinded placebo-controlled food challenge (DBPCFC)—the gold standard diagnostic test—is therefore often required.^2, 3, 7^ DBPCFC is however costly, technically challenging, time-consuming, labour-intensive, and associated with important safety risks as the procedure can trigger anaphylaxis.^2, 3, 8^ Nevertheless, although risky, DBPCFCs offer a reliable diagnosis, which in turn increases patients’ quality of life.^9^

The limitations of conventional methods for diagnosing food allergy have stimulated promising developments in molecular-based diagnostic techniques, collectively referred to as component-resolved diagnosis (CRD).^2, 8, 10^ Molecular diagnosis of food allergy involves examining the specific proteins in foods, primarily at the molecular level.^8^ In this case, rather than providing a summary of all the allergen proteins in a particular food (e.g., peanuts), molecular-based methods quantify the individual proteins within a food that may be responsible for allergic reactions.^8^ In CRD, specific IgE responses are evaluated against individual allergenic molecules or the epitopes of those allergens. Therefore, CRD techniques have the potential to enhance the specificity and sensitivity of serum-specific IgE responses to foods. They may also: facilitate our ability to determine specific food allergy phenotypes; help improve compilation of a patient-tailored risk profile to specific food allergens, given that IgE antibodies to food molecules vary from patient to patient and also geographically; and boost the ability to distinguish between primary and secondary sensitisers.^2, 8, 10^ Importantly, in CRD approaches, diagnosis can be undertaken either in single test formats or in a microarray by simultaneously evaluating a range of allergens.^2, 8, 10^

There is now a substantial body of evidence on using CRD to diagnose food allergy, which needs to be synthesised in order to generate robust estimates of diagnostic accuracy and assess cost-effectiveness in comparison with conventional approaches. Furthermore, a synthesis of the underlying evidence will help to inform deliberations on whether and how CRD should be deployed for (i) diagnosing food allergy, and (ii) assessing patient risk through the prediction of food allergy severity (i.e., the likelihood of life-threatening anaphylaxis as opposed to relatively mild features) across differing healthcare service contexts.

## Aims

We seek to identify, critically appraise, and undertake meta-analyses of studies using CRD for the diagnosis of food allergy. Specifically, we aim to:

- Determine the accuracy (i.e., sensitivity, specificity, positive and negative predictive values) of CRD for diagnosis of cow’s milk, wheat, egg, peanut, soy, tree nuts, fish, and shellfish allergy.
- Summarise the evidence on the cost and cost-effectiveness of CRD in comparison with conventional techniques for the diagnosis of cow’s milk, wheat, egg, peanut, soy, tree nuts, fish, and shellfish allergy.
- Summarise the evidence on the ability of CRD to predict the severity of cow’s milk, wheat, egg, peanut, soy, tree nuts, fish, and shellfish allergy.

## Methods

The Preferred Reporting Items for Systematic Review and Meta-Analysis Protocols (PRISMA-P) checklist has been used to guide the reporting of this protocol.^11^

### Study eligibility criteria

#### Types of studies

We will include prospective, retrospective, and cross-sectional studies. Studies must have sufficient data to calculate the relevant diagnostic measures, including sensitivity, specificity, and positive and negative predictive values. All studies must have a defined study population, with either consecutive or random sampling of participants. If the recruitment technique used to select participants is not indicated in a study, we will contact the authors to request further information about their study. In the case we do not receive a response from authors, we will include such study and undertake relevant sensitivity analyses to assess their impact on the overall test performance.

#### Types of participants

We will include studies that have examined patients with suspected food allergy of any age.

#### Target conditions

We will include studies examining the CRD diagnostic accuracy of allergenic molecules of the eight most common IgE-mediated food allergies, namely, cow’s milk, egg, wheat, soy, peanut, tree nuts (including, but not limited to hazelnut, walnut, cashew, brazil nut, pistachio, almond), fish (including, but not limited to cod and carp), and shellfish (shrimp).

#### Index test

We will include studies that have utilised molecular diagnostic techniques either based on allergenic molecules (using CRD) or the epitopes of those allergens (using epitope mapping or profiling).

#### Reference standard

We will use the DBPCFC as the gold standard for evaluating test accuracy. We will include studies in which DBPCFC has been undertaken in at least 50% of subjects in order to provide sufficient sample to strengthen the internal validity of each study. Secondarily, with regard to evaluating impact on decision-making, we will also compare CRD performance with current standard practice (i.e., clinical assessment plus SPT and/or specific IgE).

#### Exclusion criteria

We will exclude the following reports: reviews, discussion papers, non-research letters and editorials; qualitative studies; case studies, case series; animal studies; and studies that include participants on the basis of a positive food allergy test result.

### Search strategy

We will search the following databases from 1 January 2000 to 31 July 2016 for studies of diagnostic tests for food allergy: AMED (Ovid), CAB Abstracts (Ovid), the Cochrane Library, CINAHL (EBSCO), Embase (Ovid), Global Health (Ovid), MEDLINE (Ovid), PsycINFO (Ovid), Web of Science Core Collection (Thomson Reuters), WHO’s Global Health Library, and the Health Economic Evaluations Database. Although CRD methods for food allergy were already described in the 1990s,^12^ their application for food allergy diagnosis came into force fully in the 2000s, hence we have chosen the beginning of 2000 as a reasonable starting time for the literature search. We will obtain additional references by searching the references cited in identified studies, by contacting international experts and authors who have published in the field (we have compiled a preliminary list of experts to contact), and by searching the ISI Conference Proceedings Citation Index. We will search trial registries, such as Current Controlled Trials (http://www.controlled-trials.com), ClinicalTrials.gov (http://www.clinicaltrials.gov), the Australian and New Zealand Clinical Trials Registry (http://www.anzctr.org.au), and WHO’s International Clinical Trials Registry Platform (http://www.who.int/ictrp/en/) to identify ongoing studies. Using the Ovid interface for MEDLINE, we have developed a search strategy (Appendix 1) to identify and retrieve relevant studies for the review and this will be adapted in searching the other databases. There are no language restrictions for included studies: literature in languages other than English will be translated where possible, and any literature that we are unable to translate will be reported.

### Data management and selection process

We will export retrieved records from all databases to Endnote Library for study screening, de-duplication, and management of the retrieved records. Titles and abstracts of retrieved records will be screened and full text copies of potentially eligible studies will be assessed by two independent reviewers; a third reviewer will arbitrate any discrepancies.

### Data extraction

We will develop and pilot a data extraction form that will be used to collect relevant data from eligible studies. Two reviewers will independently extract relevant study data from included studies onto the form; a third reviewer will arbitrate any discrepancies.

### Data items

We will produce descriptive tables to tabulate and summarise relevant data from included studies. We will capture the following minimum set of data items from included studies: study author, country of study, year of publication, type of study design, study size, source of study population, type of food allergy, type of CRD used to diagnosed food allergy, key summary results of diagnostic accuracy, any reported risk assessment measures. For cost-effectiveness analyses, the following minimum set of data items will be extracted: competing tests analysed, economic study design type, economic perspective, test cost, measured outcome and its value, and future costs of the outcome and the test. We will use the PRISMA checklist to guide the reporting of the systematic review.^13^

### Quality assessment of studies

Risk of bias in eligible studies will be assessed by two reviewers; a third reviewer will arbitrate any discrepancies. We will assess the quality of included studies using the Quality Assessment of Diagnostic Accuracy Studies (QUADAS-2) tool.^14^ We will quality assess the economic studies using the Drummond criteria.^15^ For all studies, we will quality assess the following components of each study: patient population; index test used and interpretation; type of reference standard used; and data analysis. The quality of each of these components will be graded as high, moderate, or low.

### Data syntheses

As a first step of synthesising the evidence from included studies, we will graph the diagnostic test accuracy of the individual studies. The paired results for sensitivity and specificity will be plotted as receiver operating characteristic (ROC) curves, using these to highlight the covariation between the sensitivity and specificity in the individual studies. These initial graphical representations will also enable visual assessment of the variation between studies and facilitate subgroup analyses for exploring the effect of certain characteristics (e.g., sex, age, country/geographical region) on test performance. Expectedly, the cut-offs used to define test positivity may vary across studies; therefore, to summarise and compare the accuracy of the test across studies for each endpoint, we will employ the hierarchical summary ROC (HSROC) model.^16^ Through the inclusion of random effects, the HSROC model accounts for the variability across studies. The model uses study-specific estimates of the true positive rate (sensitivity) and the false positive rate (1—specificity) to estimate a summary ROC curve. Where studies have used a common or similar cut-off and have analysed populations with similar characteristics, we will use parameter estimates from the models to compute summary sensitivities and specificities with 95% confidence intervals. We will investigate between-study heterogeneity with the HSROC model using the following covariates to evaluate the association between study (type of study design, sampling methods employed, country of origin, cut-offs for test positivity) and patient characteristics (age, sex, disease severity) and test performance. The results from the quality assessment will be used to undertake sensitivity analyses by evaluating whether the quality of studies (low, medium, high quality) has any impact on the overall conclusion. All analyses will be performed with Stata version 14.0 (StataCorp LP, College Station, TX, USA). A narrative synthesis of the data will also be undertaken and will be the approach particularly employed in summarising the evidence pertaining to risk prediction and cost-effectiveness.

### Publication bias

We will evaluate publication bias by using funnel plots and Begg and Egger tests.^17, 18^

### Evaluating confidence in the overall evidence

We will evaluate the strength and quality of the overall evidence by using the GRADE (Grading of Recommendations Assessment, Development and Evaluation) approach.^19^ The GRADE system is useful for evaluating the quality of evidence and strength of recommendations.^19^

### Protocol registration

A detailed protocol for the review will be registered with the International Prospective Register of Systematic Reviews (PROSPERO): http://www.crd.york.ac.uk/prospero/ prior to commencing the review.

## Discussion

The major allergens in different food allergies have now been measured using the CRD technique across different settings.^8, 10, 20–23^ For example, Ara h 2 for peanut allergy, omega-5-gliadin for wheat allergy, rGly m 4 for soy milk allergy, and a number of tree nuts, fruits, vegetables, and fish have now been established.^8, 10, 20–23^ Despite the potential of CRD in improving diagnosis of food allergy, a number of important questions regarding its clinical application remain. These include a clear determination of whether these tests offer benefit over-and-above existing procedures and, if so, where in the clinical pathway CRD fits—whether as a screening tool or confirmatory method; an assessment of the impact of CRD diagnosis on the risk profile of patients; and a clear appreciation of the cost implications for its use in clinical practice in comparison with current approaches. Addressing these questions will provide a strong basis for deciding on the significance of CRD for the diagnosis of food allergy in the clinical setting. This is the first systematic review of the diagnostic accuracy of CRD for food allergy: in addition to synthesising the evidence on the diagnostic measures of the eight most common food allergies (cow’s milk, egg, wheat, soy, peanut, tree nuts, fish, and shellfish), we will, where possible, undertake risk assessment of CRD on patients, and summarise the estimates of the cost-effectiveness of CRD for diagnosing these food allergies.

## Electronic supplementary material

Appendix 1
