# Supplementary material for: Investigating the accuracy, risk impact, and cost-effectiveness of component-resolved diagnostic test for food allergy: a systematic review protocol
Source: NPJ Prim Care Respir Med. 2017 Feb 9;27:10. doi: 10.1038/s41533-017-0015-0 (PMC5434809; doi:10.1038/s41533-017-0015-0)
Supplement: Supplementary file 1 — Appendix 1 [file 41533_2017_15_MOESM1_ESM.docx]

**Appendix 1: OVID MEDLINE Search Strategy**

In developing this search strategy, we have combined relevant terminologies for food allergy and diagnostic technology, but have not included methodological terminology (study designs and economic components) so as not to restrict the scope of the search thereby potentially excluding relevant studies. The food allergy terms include common names of the food allergens, their full Latin name, and their Latin abbreviated component names.

1. exp Food Hypersensitivity/

2. food hypersensitivit*.mp.

3. food allerg*.mp.

4. allergy, food.mp.

5. Peanut Hypersensitivity/

6. Arachis/ or Peanut*.mp. or PArachis hypogaea.mp. or Ara h.mp.

7. Soybeans/ or Soy* bean.mp. or Glycine max.mp. or Gly m.mp.

8. Nuts/ or Nut Hypersensitivity/

9. Corylus/ or Hazelnut*.mp. or Corylus avellana.mp. or Cor a.mp.

10. Juglans/ or Walnut*.mp. or Juglans regia.mp. or Jug r.mp.

11. Anacardium/ or Cashew*.mp. or Anacardium occidentale.mp. or Ana o.mp.

12. Bertholletia/ or Brazil Nut*.mp. or Bertholletia excelsa.mp. or Ber e.mp.

13. Pistacia/ or Pistachio*.mp. or Pistacia vera.mp. or Pis v.mp.

15. Prunus dulcis/ or Almond*.mp. or Prunus dulcis.mp. or Pru du.mp.

16. Wheat Hypersensitivity/

17. Triticum/ or Wheat.mp. or Triticum aestivum.mp. or Tri a.mp.

18. Egg Hypersensitivity/

19. exp Eggs/ or Hen* egg*.mp.

20. Chickens/ or Chicken*.mp. or Gallus domesticus.mp. or Gal d.mp.

21. Milk Hypersensitivity/

22. Milk/ or exp Milk Proteins/ or Milk, Human/

23. Cattle/ or Cow*.mp. or Cow* milk.mp. or Bos domesticus.mp. or Bos d.mp.

24. exp Fishes/ or exp Fish Proteins/ or Parvalbumins/ or Fish allergen*.mp.

25. Penaeidae/ or Shrimp*.mp. or Penaeus aztecus.mp. or Pen a.mp. or Tropomyosin.mp.

26. exp Gadiformes/ or Cod.mp. or Gadus morhua.mp. or Gad c.mp. or Gad m.mp.

27. exp Carps/ or Carp.mp. or Cyprinus carpio.mp. or Cyp c.mp.

28. Pathology, Molecular/

29. exp Epitopes/

30. molecular diagnos*.mp.

31. molecular allerg*.mp.

32. immunoCap.mp.

33. component*.mp.

34. allergen component*.mp.

35. allergen component* diagnos*.mp.

36. molecular allergen test*.mp.

37. allergen* molecule*.mp.

38. component-resolved diagnos*.mp.

39. 1 or 2 or 3 or 4 or 5 or 6 or 7 or 8 or 9 or 10 or 11 or 12 or 13 or 14 or 15 or 16 or 17 or 18 or 19 or 20 or 21 or 22 or 23 or 24 or 25 or 26 or 27

40. 28 or 29 or 30 or 31 or 32 or 33 or 34 or 35 or 36 or 37

41. 39 and 40

42. limit 41 to yr="2000-2016"

43. Animals/ not Humans/

44. 42 not 43
